# Oral administration of linoleic acid immediately before glucose load ameliorates postprandial hyperglycemia

**DOI:** 10.3389/fphar.2023.1197743

**Published:** 2023-07-31

**Authors:** Yuta Yamamoto, Katsuya Narumi, Naoko Yamagishi, Toshio Nishi, Takao Ito, Ken Iseki, Masaki Kobayashi, Yoshimitsu Kanai

**Affiliations:** ^1^ Department of Anatomy and Cell Biology, Graduate School of Medicine, Wakayama Medical University, Wakayama, Japan; ^2^ Laboratory of Clinical Pharmaceutics and Therapeutics, Division of Pharmasciences, Faculty of Pharmaceutical Sciences, Hokkaido University, Sapporo, Japan

**Keywords:** linoleic acid, hyperglycemia, GPR40, GPR120, gastric empty, GLP-1

## Abstract

**Introduction:** Fatty acids are a major nutrient in dietary fat, some of which are ligands of long-chain fatty acid receptors, including G-protein-coupled receptor (GPR) 40 and GPR120. Pretreatment with GPR40 agonists enhanced the secretion of insulin in response to elevating blood glucose levels after glucose load in a diabetes model, but pretreatment with GPR120 agonist did not ameliorate postprandial hyperglycemia. This study examined whether oral administration of linoleic acid (LA), a GPR40 and GPR120 agonist, immediately before glucose load would affect the elevation of postprandial blood glucose levels in rats.

**Methods:** Male rats and rats with type 1 diabetes administered streptozocin were orally administered LA, trilinolein, α-linolenic acid (α-LA), oleic acid, TAK-875, or TUG-891 immediately before glucose load. Blood glucose levels were measured before, then 15, 30, 60 and 120 min after glucose load. CACO-2 cells were used to measure the uptake of [^14^C] α-MDG for 30 min with or without LA. Gastric content from rats administered LA was collected 15 and 30 min after glucose load, and blood samples were collected for measurement of glucagon-like peptide 1 (GLP-1) and cholecystokinin concentrations.

**Results:** The elevation of postprandial blood glucose levels was slowed by LA but not by trilinolein in rats without promotion of insulin secretion, and this effect was also observed in rats with type 1 diabetes. The uptake of α-MDG, an SGLT-specific substrate, was, however, not inhibited by LA. Gastric emptying was slowed by LA 15 min after glucose load, and GLP-1, but not cholecystokinin, level was elevated by LA 15 min after glucose load. TUG-891, a GPR120 agonist, ameliorated postprandial hyperglycemia but TAK-875, a GPR40 agonist, did not. Pretreatment with AH7614, a GPR120 antagonist, partially canceled the improvement of postprandial hyperglycemia induced by LA. α-LA, which has high affinity with GPR120 as well as LA, slowed the elevation of postprandial blood glucose levels, but oleic acid, which has lower affinity with GPR120 than LA, did not.

**Conclusion:** Oral administration of LA immediately after glucose load ameliorated postprandial hyperglycemia due to slowing of gastric emptying via promotion of GLP-1 secretion. The mechanisms may be associated with GPR120 pathway.

## 1 Introduction

Diet is an important factor in maintaining energy homeostasis, and it is taken orally by animals. Fat, a major nutrient within a diet, is digested into fatty acids and glycerol by pancreatic lipase in the small intestine (Porsgaard et al., 2005). Fatty acid is absorbed as nutrition in the small intestine, and particular fatty acids are ligands of fatty acid receptors which detect concentration of fatty acid in the gastrointestinal tract to secrete hormones in intestinal mucosa (Yu et al., 2020). Long chain fatty acids are major fatty acids digested from dietary fat, and some long chain fatty acids are ligands of the fatty acid-specific G-protein-coupled receptors (GPRs), such as GPR40 and GPR120 (Briscoe et al., 2003; Itoh et al., 2003; Hirasawa et al., 2005; Briscoe et al., 2006; Kimura et al., 2020).

GPR120 is expressed in endocrine cells from the small intestine to the large intestine (Anbazhagan et al., 2016). In mice, a high-fat diet containing fatty acids including GPR120 ligands for 5 weeks reportedly reduced insulin resistance through the inhibitory effect of transforming growth factor-β activated kinase 1 (Oh et al., 2010). GPR120 is expressed in the enteroendocrine cells, and STC-1 cells, an enteroendocrine cell line, secrete glucagon-like peptide 1 (GLP-1) and cholecystokinin (CCK) in response to long chain fatty acid recognized by GPR120 (Hirasawa et al., 2005; Tanaka et al., 2008). Oral pre-administration of GPR120 ligand (TUG-891) tends to ameliorate hyperglycemia after glucose load in rats (Li et al., 2017). GPR40 is highly expressed in the ileum, especially in L cells, which secrete GLP-1 (Edfalk et al., 2008; Little et al., 2014). Beta cells in the human pancreas express GPR40 (Tomita et al., 2005) and GLP-1 receptor, which enhance the secretion of insulin in response to the elevation of blood glucose level (Luo et al., 2012). In rats, the oral administration of GPR40 ligand, fasiglifam (TAK-875) and MR1704 an hour or more before glucose load reportedly decreased the postprandial glucose level after the glucose load by promoting insulin secretion in response to elevation of blood glucose level (Tsujihata et al., 2011; Tsuda et al., 2017). Long-term administration of GPR120 agonists and pre-administration of GPR40 agonists is said to allow the possibility of development of drugs for type 2 diabetes via insulin-dependent pathway (Christiansen et al., 2008; Tikhonova et al., 2008; Oh et al., 2010). However, when administered immediately before glucose load, the effects of GPR40 and GPR120 agonists in rodents or humans have been unclear.

Gastric emptying is controlled by various mechanisms including the endocrine system and/or nervous system, and it regulates the sending of gastric content in the gastric cavity to the small intestine (Goyal et al., 2019). Postprandial glycemia is attenuated by gastric emptying being slowed in healthy subjects (Deane et al., 2010). Endocrine cells in the small intestine sense the nutritional environment of their cavity, and they secrete CCK and GLP-1, which slows the gastric emptying (Deane et al., 2010; Kimura et al., 2020). Linoleic acid (LA), a dual agonist of GPR40 and GPR120, is of the triglyceride type in nature and is contained in vegetable oils, including olive oil. However, LA contained in triglycerides might not reveal the agonist activity of GPR40 and GPR120 immediately after taking triglycerides containing LA. This is because agonist activity of GPR40 and GPR120 is revealed in LA as a fatty acid, but not LA contained in triglycerides. Moreover, time is needed to digest the triglyceride from the daily diet to fatty acid and glycerol in the duodenum by pancreatic lipase. In this sense, oral administration of LA as fatty acid was different from that of LA contained in triglyceride regarding the immediacy of the effect. Oral administration of LA, a free fatty acid, appears to promote GLP-1 and/or CCK secretions immediately after LA administration. This could result in slowing the elevation of postprandial blood glucose level.

This study investigates whether the oral administration of LA taken immediately before glucose load slows the elevation of postprandial blood glucose level. We also examine whether the beneficial effect is induced by GLP-1 or CCK, and whether their secretions are associated with the GPR40 or GPR120 pathway.

## 2 Materials and methods

### 2.1 Animals

Five-week-old male Sprague–Dawley (SD) rats were purchased from CLEA Japan, Inc. (Tokyo, Japan). Two or three rats were housed in a plastic rat cage (24.7 cm × 40.9 cm × 19.7 cm) with free access to tap water and laboratory animal feed (Oriental Yeast Co., Ltd., Tokyo, Japan) under a 12 h light/dark cycle (lights on/off at 8:00 a.m./p.m.) at 25°C ± 1°C and 50%–60% humidity. All animals were used for experiments after an acclimation period of 1 week. The experimental protocol was approved by the Wakayama Medical University Animal Experiment Committee (Nos 883 and 1050).

### 2.2 Chemicals

The following chemicals were purchased: LA (L0124, Tokyo Chemical Industry, Tokyo, Japan), trilinolein (T1388, Tokyo Chemical Industry), oleic acid (25630-64, Nacalai Tesque, Kyoto, Japan), α-linolenic acid (122-05831, FUJIFILM Wako Pure Chemical Corp., Osaka, Japan), streptozocin (195-15154, FUJIFILM Wako Pure Chemical Corp), olive oil (7121704X1407, Yoshida Pharmaceutical Company, Tokyo, Japan), [^14^C]-Labelled methyl-α-D-glucopyranoside ([^14^C] α-MDG; 50 μCi; 1.85 MBq; 250 mCi/mmol) (NEC659V, Perkin Elmer, Waltham, MA, United States), phlorizin (167-24401, FUJIFILM Wako Pure Chemical Corp.), TAK-875 (HY-10480, MedChemExpress, Monmouth Junction, NJ, United States), TUG-891 (HY-100881, MedChemExpress), and AH7614 (ab146181, Abcam, Cambridge, United Kingdom).

### 2.3 Type 1 diabetes model rats

Type 1 diabetes (T1DM) rats were made according to a modified version of a protocol used in a previous study (Luippold et al., 2012). It was modified because six-week-old male SD rats could not be deprived in insulin secretion after glucose load as in the previous study, which used eight or nine-week-old male SD rats. Six-week-old male SD rats were treated with dual intraperitoneal dose of 60 mg/kg streptozocin on the first day and 100 mg/kg streptozocin on the second day. In the following week, rats were loaded with glucose (2 g/kg body weight), and blood samples were collected before or 30 min after glucose load. Rats were considered to have T1DM under the condition of blood glucose concentration 30 min after glucose load being >500 mg/dL, and the ratio of plasma insulin concentration being 30 min/0 min: > 1.5.

### 2.4 Cell culture

CACO-2 cells were purchased from Deutsche Sammlung für Mikroorganismen und Zellkulturen (Braunschweig, Germany) and were cultured in Dulbecco’s modified Eagle’s medium supplemented with 10% fetal bovine serum, 1% non-essential amino acids, and 100 IU/mL penicillin-100 μg/mL streptomycin. Cells were grown in an atmosphere of 5% CO_2_-95% air at 37°C.

### 2.5 [^14^C] α-MDG uptake study

CACO-2 cells were seeded at a cell density of 2.0 × 10^5^ cells/well on 24-well plastic plates. The medium of the cell monolayers was refreshed every 2 days, and the monolayers were used for the uptake experiments on day 14 after plating. After removal of the culture medium, each well was washed and pre-incubated with glucose-free Hank’s balanced salt solution (HBSS) buffer (137 mM NaCl, 5.37 mM KCl, 0.3 mM Na_2_HPO_4_, 0.44 mM KH_2_PO_4_, 1.26 mM CaCl_2_, 0.8 mM MgSO_4_, 4.17 mM NaHCO_3_, and 10 mM HEPES; pH 7.4 adjusted with 1 M Tris). Then, 0.5 mL of HBSS buffer or one replaced from 137 mM NaCl to 137 mM choline chloride which contained 0.4 μM (0.1 μCi/mL) [^14^C] α-MDG with compounds (50 μM LA, 100 μM LA or 100 μM phlorizin) were used as incubated medium. CACO-2 cells were incubated at 37°C for 30 min when the uptake of α-MDG in HBSS with Na^+^ was remarkably different from one in HBSS without Na^+^ (Supplementary Figure S1). Each cell monolayer was rapidly washed twice with 1.0 mL ice-cold HBSS buffer at the end of the incubation period. To quantify the radioactivity of [^14^C] α-MDG, the cells were solubilized in 1% SDS/0.2 N NaOH. The remainder of the sample was mixed with 3 mL of scintillation cocktail to estimate the radioactivity. All uptake values were corrected for protein content and the protein concentration was determined using a Pierce^®^ BCA Protein Assay Kit (Thermo Fisher Scientific, Waltham MA, United States). The difference of α-MDG uptake between HBSS with Na^+^ and HBSS without Na^+^ was the α-MDG uptake via SGLT1. The experiments were performed three times.

### 2.6 Oral glucose tolerance test

Five rats were allocated to each group, and we examined oral glucose tolerance test (OGTT) in the normal rats at 6 weeks of age. OGTT was performed by oral administration of glucose solution (2 g/kg body weight). Just before the glucose load, rats fasted overnight were orally administered one of the oil solutions (each 5 mL/kg body weight). Oil solutions were either olive oil, LA dissolved with olive oil (2.5 g/mL), trilinolein dissolved with olive oil (2.5 g/mL), oleic acid dissolved with olive oil (2.5 g/mL), or α-linolenic acid dissolved with olive oil (2.5 g/mL). Blood samples were collected from the tail vein before administration of the oils and then 15, 30, 60, and 120 min after glucose load. Blood glucose levels were directly measured in blood samples with Freestyle Libre (Abbott, Alameda, CA). After measuring blood glucose levels, plasma samples were collected from the tail vein and added to aprotinin (final concentration 250 KIU/mL), and plasma insulin, glucagon-like peptide 1 (GLP-1) and cholecystokinin (CCK) levels were measured using rat insulin measurement kit (Morinaga Institute of Biological Science, Kanagawa, Japan), and rat GLP-1 ELISA kit (FUJIFILM Wako Pure Chemical Corporation) rat CCK Enzyme Immunoassay Kit (RayBiotech, Norcross, GA). T1DM rats were subjected to OGTT at 8 weeks of age. Blood glucose levels were directly measured in blood samples from the tail with Freestyle Libre before the administration of oils, and then at 15, 30, 60, and 120 min after glucose load.

To measure the amount of glucose in the gastric tract, we collected stomachs ligated at the cardia and the pylorus from anesthetized rats with isoflurane 15 or 30 min after glucose load following blood glucose level in portal vein with Freestyle Libre, and then collected gastric content in cold phosphate-buffered saline. The volume of the supernatant was measured after centrifuging gastric content with phosphate-buffered saline at 400 g for 10 min, and the glucose concentration in the supernatant was measured with Lab Assay Glucose (FUJIFILM Wako Pure Chemical Corporation). The amount of glucose in the gastric tract was calculated from the volume and concentration. Residual ratio of glucose in the stomach was calculated from the ratio of the amount of glucose in the gastric tract to the amount of orally-administered glucose.

To analyze the effect of GPR40 or GPR120 agonists, rats fasted overnight were orally administered either TAK-875 suspension (5 and 10 mg/kg body weight) or TUG-891 suspension (20 mg/kg body weight) in olive oil immediately before glucose load or 60 min before glucose load. Dose of TAK-875 was 1, 3 and 10 mg/kg, and dose of TUG-891 was 30 mg/kg, as in previous studies (Tsujihata et al., 2011). Blood glucose levels in these rats were measured in blood samples from the tail veins with Freestyle Libre.

To examine the inhibitory effect of GPR120 antagonist in rats administered LA immediately before glucose load, rats fasted overnight were orally administered AH7614 solved with 0.5% CMC solution (25 mg/kg, 2.5 mL/kg), GPR120 antagonist, 10 min before LA and glucose load. Blood glucose levels were measured in the same way as other OGTT experiments. Dose of AH7614 in this study was 28.5 mmol/L suspension, because in a previous study, 1 µM of AH7614 fully inhibited GPR120 agonistic activity of LA (Sparks et al., 2014).

### 2.7 Statistical analysis

Statistical analyses were performed using JMP 14 (SAS Institute, Cary, NC). Repeated measures analysis of variance (ANOVA) was used in analysis of blood glucose level changes after glucose load, and the significance level was 0.05. The significance level was 0.0166, calculated with Bonferroni correction in multiple comparison of three groups. Student’s *t*-test was used for two-group comparison and Tukey-HSD test for three-group comparison, and significance level was 0.05. Results are shown as means ± SDs in figures.

## 3 Results

### 3.1 Effect of linoleic acid administration immediately before glucose load on postprandial hyperglycemia

**FIGURE 1:**
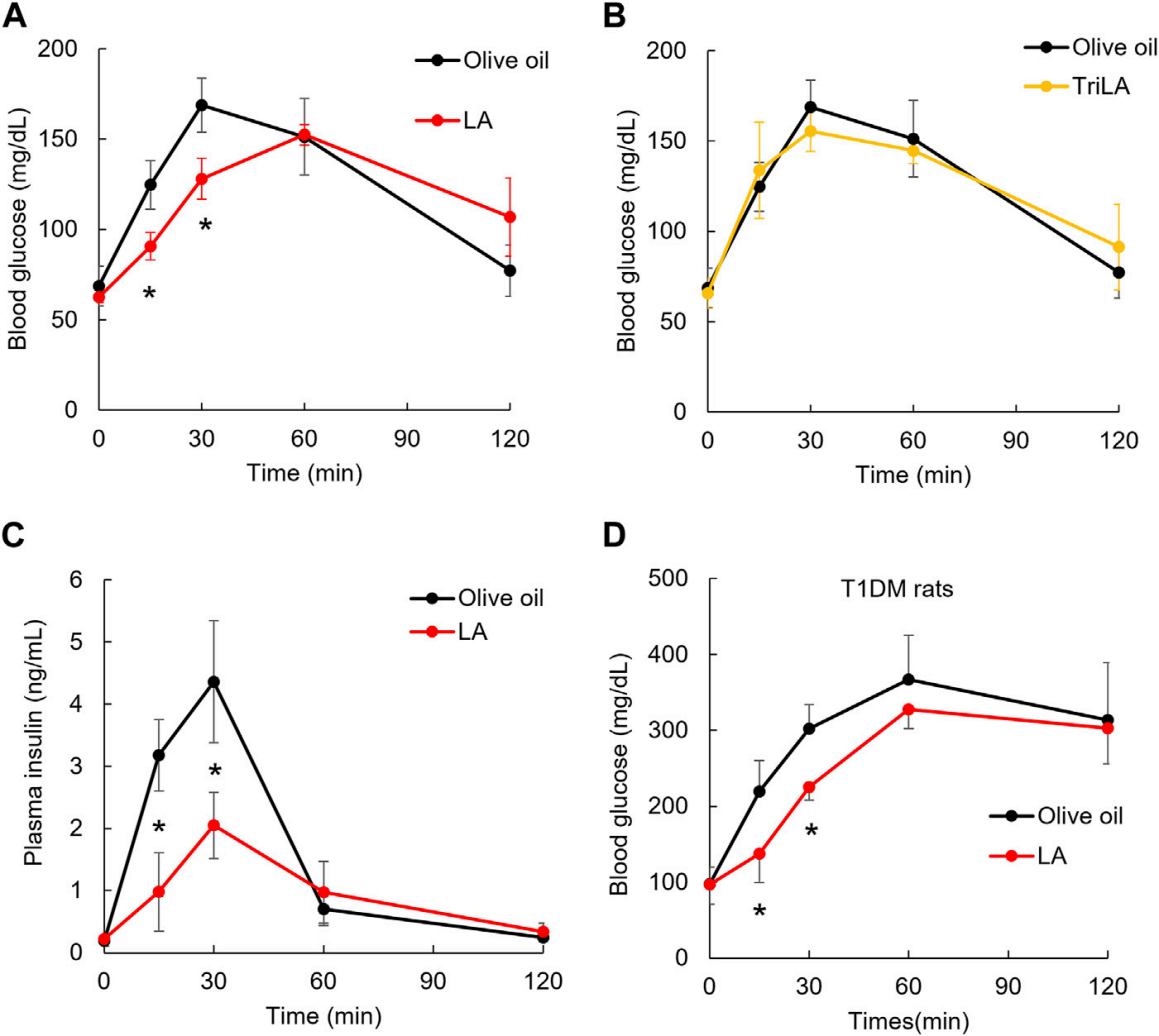
Linoleic acid slowed the elevating postprandial blood glucose level independently from insulin. LA immediately after glucose load slowed the elevation of postprandial glucose level. Oral administration of LA but not trilinolein (TriLA) slowed the elevation of postprandial glucose level **(A,B)** without enhancing secretion of insulin **(C)**. The slowing elevation of postprandial glucose level was also shown in rats with type 1 diabetes administered LA immediately before glucose load **(D)**. Asterisks indicate significant difference between olive oil and LA groups.

Focusing on the oral administration of linoleic acid immediately before glucose load, we measured the postprandial glucose levels 15, 30, 60 and 120 min after glucose load in rats orally administered LA or olive oil (Figure 1A). Repeated measures ANOVA indicated that the effect of LA on the blood glucose level was not significant [F _(1, 8)_ = 2.9, *p* = 0.13]. The effect of time was significant [F _(4, 32)_ = 109.6, *p* < 0.01] and their interaction was also significant [F _(4, 32)_ = 16.2, *p* < 0.01] (Figure 1A). The postprandial glucose levels were lower in the LA group 15 and 30 min after glucose load than in the olive oil group, but it was higher in LA group 120 min after glucose load than in the olive oil group (Figure 1A). Thus, oral administration of linoleic acid immediately before glucose load did not decrease the postprandial glucose level. LA might, however, be able to slow the elevation of the postprandial glucose levels. We statistically reanalyzed the postprandial glucose levels 30 min after glucose load with repeated measures ANOVA. The postprandial glucose levels up to 30 min after glucose load were significantly decreased in the LA group [F _(1, 8)_ = 23.2, *p* < 0.01]. We therefore examined the hypothesis that LA slowed the elevation of the postprandial glucose levels by 30 min after glucose load. The dose-dependent manner of LA administration was confirmed in our preliminary data (Supplementary Figure S2). Repeated measures ANOVA indicated that dose of LA (625 mg/kg) decreased the postprandial blood glucose level [F _(1, 8)_ = 11.2, *p* < 0.01], but dose of LA (313 mg/dL) did not decrease it [F _(1, 8)_ = 0.2, *p* = 0.64].

To examine whether there were beneficial effects of triglycerides containing LA as well as LA, rats were orally administered TriLA, which are esters formed from glycerol and LA. Repeated measures ANOVA indicated that the postprandial glucose levels up to 30 min after glucose load were not decreased in the TriLA group [F _(1, 8)_ = 0.1, *p* = 0.75] (Figure 1B). To examine whether the slowing elevation of postprandial blood glucose level 30 min after glucose load in the LA group was caused by promotion of insulin secretion, plasma insulin concentrations were measured (Figure 1C). Repeated measures ANOVA indicated that the plasma insulin concentrations were decreased in the LA group [F _(1, 8)_ = 31.0, *p* < 0.01]. We made T1DM model rats using streptozocin and measured blood glucose concentration in T1DM model rats administered LA or olive oil (Figure 1D). Repeated measures ANOVA indicated that the postprandial glucose levels up to 30 min after glucose load were significantly decreased in the LA group [F _(1, 6)_ = 8.4, *p* = 0.03] (Figure 1D).

### 3.2 LA did not inhibit the glucose intake in the intestinal tract

**FIGURE 2:**
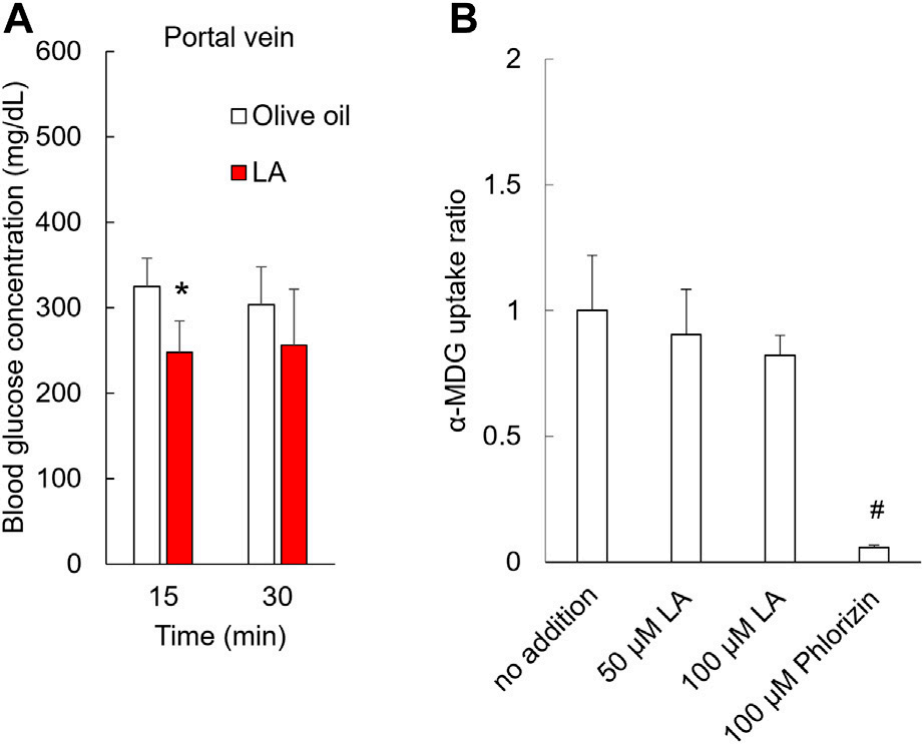
LA inhibited the absorption of glucose in the small intestine but did not inhibit SGLT1 transport. Blood glucose level in the portal vein decreased in rats administered LA **(A)**. LA did not inhibit the uptake of SGLT1 in Caco-2 cells **(B)**. Asterisk indicates significant difference between olive oil and LA groups. # indicates significant difference from no addition group.

The slowing of postprandial blood glucose level is independent from promotion of insulin secretion. We focused on the glucose absorption in the small intestine and measured the blood glucose level in the rat portal vein 15 or 30 min after glucose load. The postprandial glucose levels in the portal vein were lower in the LA group than in the olive oil group at 15 min (*p* < 0.01, Figure 2A). LA may therefore inhibit the glucose intake in mucosa of the small intestine or decrease the rate of gastric emptying.

**FIGURE 3:**
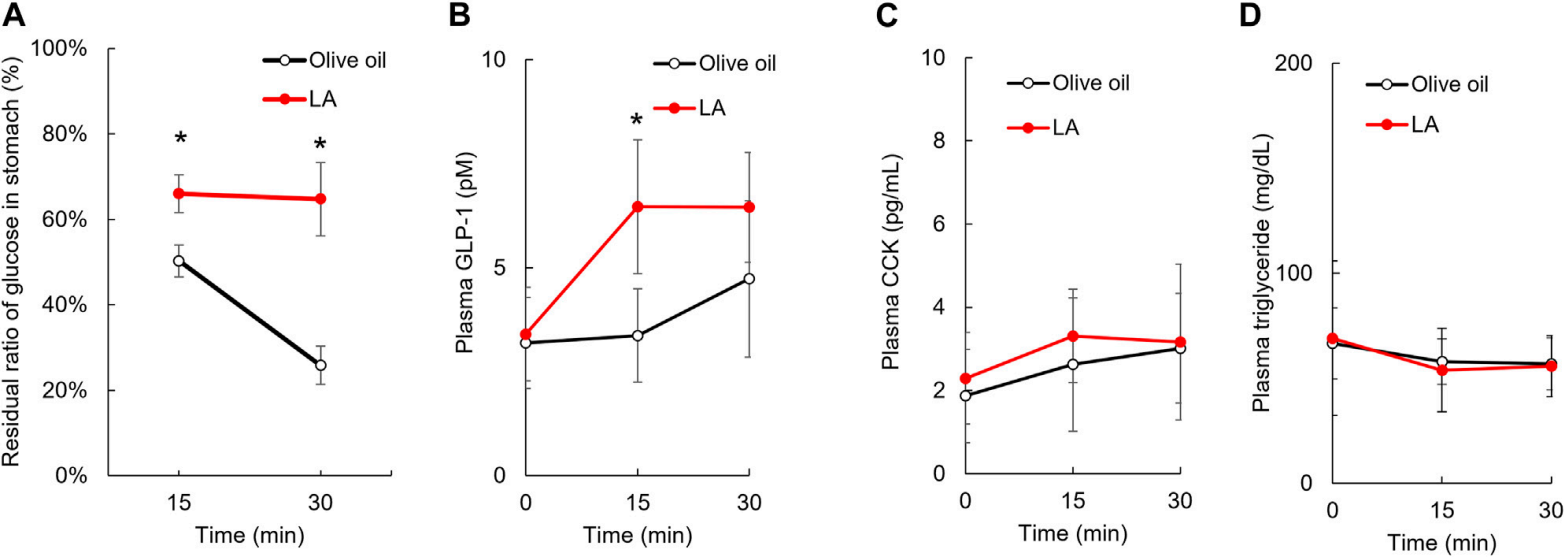
Linoleic acid decreases gastric motility in promoting GLP-1 secretion. LA inhibited the movement of loaded glucose from the stomach to the duodenum. The amount of loaded glucose in stomach did not change in rats administered LA **(A)**. Plasma concentration of GLP-1, CCK and triglyceride before and then 15 and 30 min after oil and glucose load **(B–D)**. Asterisks indicate significant difference between olive oil and LA groups.

To examine whether LA inhibited the glucose intake in sodium-dependent glucose transporter (SGLT) 1 located on the brush border membrane of intestinal epithelium cells, we measured the glucose uptake under LA or phlorizin, an SGLT inhibitor, in CACO-2 cells. Phlorizin inhibited the uptake of α-MDG, an SGLT1-specific substrate (*p* < 0.01), but LA did not affect the uptake of α-MDG (Figure 2B). Next, we measured the amount of glucose in the gastric tract 15 or 30 min after glucose load. In the olive oil group, the amounts of glucose in the gastric tract were approximately 50% or 25% of the amount of glucose administered orally 15 or 30 min after glucose load. In the LA group, however, they were approximately 65% of the amount of glucose administered orally 15 and 30 min after glucose load (Figure 3A). Student’s *t*-test indicated that the residual ratio of glucose in the stomach was significantly higher in the LA group than in the control group at both 15 min (*p* < 0.01) and 30 min (*p* < 0.01). LA had therefore already slowed gastric motility 15 min after glucose load and LA indirectly inhibited glucose intake.

Gastric motility is controlled by several hormones, including GLP-1 and CCK, which are secreted in stimulations of GPR40 and GPR120 agonists. We measured the concentration of plasma GLP-1 and CCK 15 and 30 min after glucose load to examine whether these hormones were associated with slowing gastric motility for LA. Repeated measures ANOVA indicated that the plasma GLP-1 concentrations were increased in the LA group [F _(1, 9)_ = 11.3, *p* < 0.01] (Figure 3B), but the plasma CCK concentrations were not changed in the LA group [F _(1, 10)_ = 0.9, *p* = 0.37] (Figure 3C). The plasma triglyceride concentrations were not changed in the LA group [F _(1, 10)_ = 0.0, *p* = 0.92] (Figure 3D).

### 3.3 Slowing elevation of postprandial blood glucose level was associated with GPR120 pathway

**FIGURE 4:**
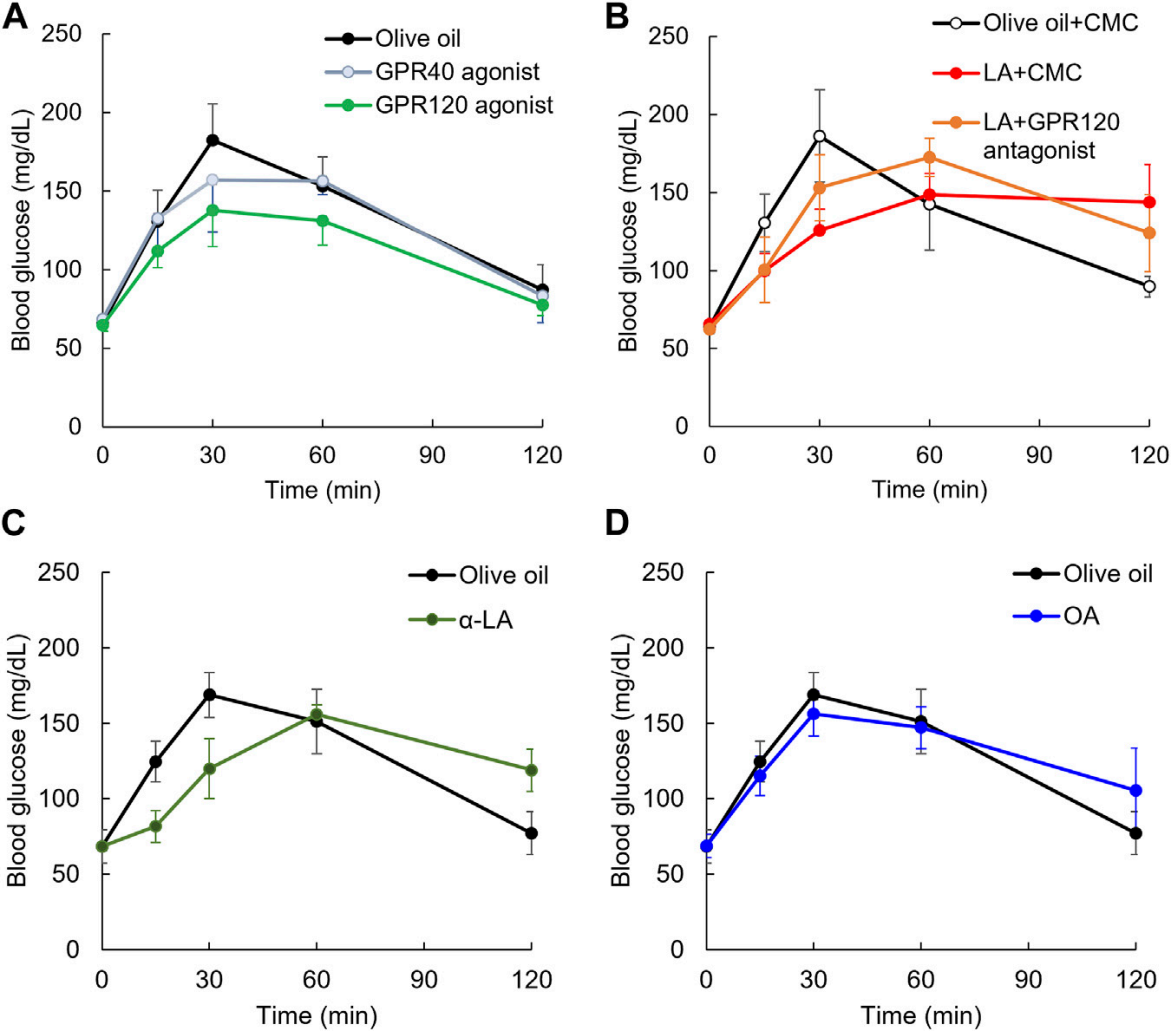
Slowing elevation of postprandial blood glucose level on GPR120 agonists. Elevating postprandial glucose level was slowed by GPR120 agonist, TUG-891, but not GPR40 agonist, TAK-875 **(A)**. Slowing elevation of postprandial blood glucose level in rats administered LA was partially canceled by pretreatment of GPR120 antagonist **(B)**. α-Linoleic acid (α-LA), GPR120 ligand as same affinity as LA, slowed the elevation of postprandial glucose level **(C)**. Oleic acid (OA), whose GPR120 ligand affinity was lower than LA, slowed the elevation of postprandial glucose level **(D)**.

The effect of GPR40 or GPR120 agonists on the postprandial blood glucose in rodents administered GPR40 or GPR120 agonist before glucose load has been previously reported (Tsujihata et al., 2011; Li et al., 2017). We examined whether the elevation of postprandial glucose levels was slowed in rats administered these agonists immediately before glucose load as well as LA. Repeated measures ANOVA indicated that TUG-891, a GPR120 agonist, decreased the elevation of postprandial glucose level by 30 min after glucose load compared with olive oil [F _(1, 12)_ = 14.3, *p* < 0.01], but TAK-875 (10 mg/kg), a GPR40 agonist, did not decrease it [F _(1, 11)_ = 0.4, *p* = 0.52] (Figure 4A). A half dose of TAK-875 (5 mg/kg) did not decrease it (Supplementary Figure S3). The elevation of postprandial glucose level up to 30 min after glucose load was not different between GPR120 and GPR40 agonist [F _(1, 11)_ = 4.2, *p* = 0.07] (Figure 4A). Furthermore, repeated measures ANOVA indicated that TUG-891 decreased the elevation of postprandial glucose level up to 120 min after glucose load compared with olive oil [F _(1, 12)_ = 24.3, *p* < 0.01].

Focusing on the GPR120 pathway, we used rats administered AH7614, a GPR120 antagonist, 10 min before glucose and LA load. The elevation of postprandial glucose levels by 30 min after glucose load was decreased by LA compared with in the olive oil group [F _(1, 8)_ = 41.6, *p* < 0.01], but it was not decreased by LA + AH7614 compared with in the olive oil group [F _(1, 8)_ = 9, *p* = 0.017] (Figure 4B). However, the elevation of postprandial glucose level by 30 min after glucose load was not different between LA and LA + AH7614 [F _(1, 8)_ = 2.1, *p* = 0.19] (Figure 4B).

To confirm the slowing elevation of postprandial glucose level induced by GPR120 ligand but not GPR40 ligand, we used α-LA, which has the same affinity with GPR120 as LA. Repeated measures ANOVA indicated that the postprandial glucose levels up to 30 min after glucose load was significantly decreased in the α-LA group compared with in the olive oil group [F _(1, 7)_ = 8.4, *p* = 0.03] (Figure 4C). We also confirmed the slowing of the elevation of postprandial glucose level induced by OA, which has lower affinity with GPR120 than LA. Repeated measures ANOVA indicated that the postprandial glucose levels up to 30 min after glucose load were not decreased in the oleic acid group [F _(1, 8)_ = 1.2, *p* = 0.30] (Figure 4D).

## 4 Discussion

Particular long-chain fatty acids are the agonist of GPR40 and GPR120, which are associated with gastrointestinal endocrine system, as well as nutrition. GPR40 agonists were previously reported to promote the secretion of insulin in response to the elevating glucose level when rats were administered the agonists one or 2 h before glucose load, and to decrease the postprandial glucose level and plasma glucose AUC _0–120 min_ after glucose load (Tsujihata et al., 2011; Tsuda et al., 2017). GPR120 agonists improved insulin resistance in long-term administration (≥8 weeks) (Oliveira et al., 2015; Jia and Liu, 2022), but did not ameliorate postprandial hyperglycemia by single oral administration 30 min before glucose load in mice (Li et al., 2017). This study indicates that LA, an agonist of both GPR40 and GPR120, slowed the elevation of postprandial blood glucose level when rats were administered LA immediately before the glucose load. This mechanism was slowing of gastric motility, and it is associated with promoting secretion of GLP-1 via GPR120 signaling.

Oral administration of LA immediately before glucose load did not decrease postprandial blood glucose levels by 120 min after glucose load, but it did decrease them up to 30 min after glucose load (Figure 1A). This effect is different from the effect of peroral administration of GPR40 agonist on postprandial glucose levels (Tsujihata et al., 2011), because the pretreatment of GPR40 agonist promotes insulin secretion in response to the elevating glucose levels, then decreases the postprandial glucose levels compared with a control. This GPR40 agonist effect may therefore be the superior to the LA effect in healthy individuals or patients with type 2 diabetes. However, oral administration of LA immediately before glucose load did not promote insulin secretion in response to the elevation of postprandial glucose level in normal rats (Figure 1C), and it decreased the postprandial glucose levels by 30 min after glucose load in the most severe T1DM model rats, whose ability to secrete insulin in response to the elevating glucose level was impaired by streptozocin (Figure 1D). LA could therefore slow the elevation of postprandial glucose level in patients with T1DM who take LA immediately before a meal. Previous clinical studies of T1DM indicated that the administration of rapid-acting insulin analogs immediately before meals resulted in the postprandial blood glucose level being higher than 180 mg/dL, and this administration 15 or 20 min before meals kept postprandial blood glucose levels under 180 mg/dL (Cobry et al., 2010; Luijf et al., 2010). Time-in-range has been proposed, it is the percentage of time patients spend with blood glucose level between 70 and 180 mg/dL (American Diabetes Association, 2021). Time-in-range has been reported to be negatively correlated with hemoglobin A1c (Valenzano et al., 2021). This administration of LA may slow the elevation of postprandial blood glucose level and extend the time when the postprandial blood glucose level is peak in patients with T1DM. The extension may be preferable to the recommended 15–20 min delay before being able to eat after the injection of insulin. Thus, administration of LA and insulin immediately before meals may improve hemoglobin A1c in T1DM. The oral administration of GPR40 agonists does not ameliorate the postprandial glucose level in patients with T1DM whose ability to secrete insulin is impaired. Future studies will examine the effect of oral administration of LA immediately before meals on the elevating postprandial glucose levels in patients with T1DM including during the honeymoon period of T1DM and in slowly progressive insulin-dependent T1DM.

We demonstrated that the oral administration of TriLA esterized with LA and glycerin immediately before glucose load and did not decrease the postprandial glucose levels up to 30 min after glucose load (Figure 1B). This result suggests that LA contained in triglycerides including olive oil and TriLA did not slow the elevation of postprandial glucose levels in rats administered them immediately before glucose load. Fatty acids, but not those in triglyceride form, are signaling molecules recognized by free fatty acids receptors including GPR40 and GPR120 (Hara et al., 2013). In order to indicate the agonist activity of GPR40 and GPR120, triglyceride taken orally is needed to be driven to the duodenum and digested by pancreatic lipase (Zoller et al., 2022). Oral administration of TriLA immediately before glucose load may therefore be insufficient to have the GPR40 and/or GPR120-agonisticeffect immediately after TriLA load. However, the interaction between LA and compounds (e.g., polyphenols, vitamin E, phytosterols and carotenoids) included in olive oil was not excluded in this study because olive oil was used as control and solvent of fatty acid and TriLA to minimize the difference of nutrition intake between the control and other groups.

Glucose is transported by SGLT 1 and 2, which are located on the brush border lumen of the small intestine and the proximal tubule (Du et al., 2018), and SGLT1 is expressed in the small intestine, especially the jejunum, but SGLT2 is not (Balen et al., 2008; Wright et al., 2011). We measured the blood glucose in the portal vein to examine whether LA also inhibited glucose uptake in the small intestine. LA decreased the postprandial glucose level in the portal vein 15 min after glucose load (Figure 2A), so LA might inhibit glucose uptake via SGLT1 in the small intestine. α-MDG is a selective substrate of SGLTs (Mitsuoka et al., 2016), and phlorizin is an antagonist of SGLT1 and SGLT2 (Niederberger et al., 2020). CACO-2 cells from DSMZ were previously shown to efficiently express SGLT1, and α-MDG uptake in CACO-2 cells from DSMZ was at maximum efficiency 13 or more days after seeding (Steffansen et al., 2017). CACO-2 cells from DSMZ were used to examine whether LA was an antagonist of SGLT1, and LA did not inhibit uptake of α-MDG (Figure 2B). LA did not therefore directly inhibit glucose uptake in lumen of the small intestine via SGLT1.

Gastric motility is the delivery of gastric contents into the duodenum and is slowed by several factors, including hormones (GLP-1 and CCK) (Goyal et al., 2019). LA might decrease the rate of gastric emptying, and we measured the remaining amount of glucose in the gastric tract. The amount of loaded glucose was reduced to 50% 15 min after glucose load, and to 30% 30 min after glucose load in control group (Figure 3A). LA had slowed this gastric motility at 15 min after glucose load, and the motility had almost disappeared between 15 and 30 min after glucose load. Oral administration of LA increased the plasma GLP-1 concentration, but not CCK (Figures 3B, C). Oral administration of LA might therefore slow gastric motility quickly via GLP-1 signaling, and inhibit movement of glucose from the stomach to the duodenum, because GLP-1 slows the gastric emptying (Deane et al., 2010). However, it was unclear whether oral administration of LA immediately before glucose load directly promoted GLP-1 secretion via GPR120 pathway and slowed gastric empty by GLP-1. Future studies should clarify this concern using GLP-1 antagonist or GPR120 knockout mice.

LA is a dual ligand of both GPR40 and GPR120 (Kimura et al., 2020), but previous studies indicated the effect of postprandial glucose level on pretreatment of GPR40 or GPR120 agonists (Tsujihata et al., 2011; Li et al., 2017). It was unclear, however, whether the postprandial glucose level was improved by administration of these agonists immediately before glucose load. TUG-891, a GPR120 agonist, decreased the postprandial glucose level by 30 min after glucose load compared with the olive oil group, but GPR40 agonist did not show beneficial effects (Figure 4A). Pretreatment of GPR120 antagonist partially canceled the beneficial effects induced by LA (Figure 4B). Alpha-LA has high affinity of ligands in both GPR40 and 120, but oleic acid had high affinity of ligand in GPR40 and not in GPR120 (Kimura et al., 2020). The oral administration of α-LA immediately before glucose load slowed the elevation of postprandial glucose level, but oleic acid did not reveal the beneficial effects (Figures 4C, D). Slowing the elevation of postprandial glucose level in oral administration of LA may therefore be associated with GPR120 signaling.

This study used olive oil as control and solvent of fatty acids or TriLA to minimize the difference of nutrition intakes. However, olive oil contains polyphenols, vitamin E, phytosterols, and carotenoids derived from olives as well as triglyceride, and these substances might affect the postprandial glucose levels. In this study, the solvent in the LA (625 mg/kg) group containing 87.5% olive oil and 12.5% LA also slowed the postprandial glucose levels (Supplementary Figure S2), but the solvent in the TriLA group containing 50% olive oil and 50% TriLA did not slow the postprandial glucose levels (Figure 1B). Thus, the concern was almost minimized, but was not fully solved in this study.

This study indicated that the oral administration of GPR120 agonist immediately before glucose load decreased the postprandial blood glucose levels up to 120 min after glucose load (Figure 4A). In case of LA administration, the blood glucose level 120 min after glucose load was high compared with in the olive oil group. The higher blood glucose levels were also observed in α-LA and oleic acid group but not in the olive oil or the TriLA group (Figures 1A, B, 4C, D). This maintained higher glucose level was also observed in TAK-875 group in a dose-dependent manner (Supplementary Figure S3). Thus, the oral administration of fatty acids immediately before glucose load might therefore inhibit decrease of the postprandial glucose level 120 min after glucose load, and this mechanism might be associated with GPR40 pathway. TUG-891, a GPR120 agonist, did not indicate this effect, so a GPR120 agonist could ameliorate the postprandial hyperglycemia. The EC50 of GPR120 is 75.3 nM, and that of GPR40 is 52.6 μM in TUG-891 (Li et al., 2017). The EC50 of GPR120 is more than 10 μM, and that of GPR40 is 33.6 nM in TAK-875 (Li et al., 2017). The EC50 of GPR120 is 1 μM, and that of GPR40 is 2 μM in LA (Kimura et al., 2020). These selective GPR120 or GPR40 agonists have 10 or 20-fold affinity with each receptor compared with LA. Thus, GPR120 agonist, which has high affinity and selectivity of GPR120, might decrease postprandial glucose levels in animals and humans immediately before glucose load or meals. Further studies will examine the oral administration of selective GPR120 agonists which decrease the postprandial blood glucose level immediately before glucose load in a dose-dependent manner, with the ultimate aim of developing drugs for individuals with type 1 as well as type 2 diabetes.

Oral administration of free LA, a GPR120 agonist, immediately before glucose load improved postprandial glucose levels independent from insulin. LA contained in dietary fat may therefore improve postprandial hyperglycemia in patients with T1DM.

## Data Availability

The raw data supporting the conclusion of this article will be made available by the authors, without undue reservation.
