# Supplementary material for: Oral administration of linoleic acid immediately before glucose load ameliorates postprandial hyperglycemia
Source: Front Pharmacol. 2023 Jul 31;14:1197743. doi: 10.3389/fphar.2023.1197743 (PMC10424117; doi:10.3389/fphar.2023.1197743)
Supplement: Supplementary file 1 [file Presentation1.pdf]

Supplementary Figures

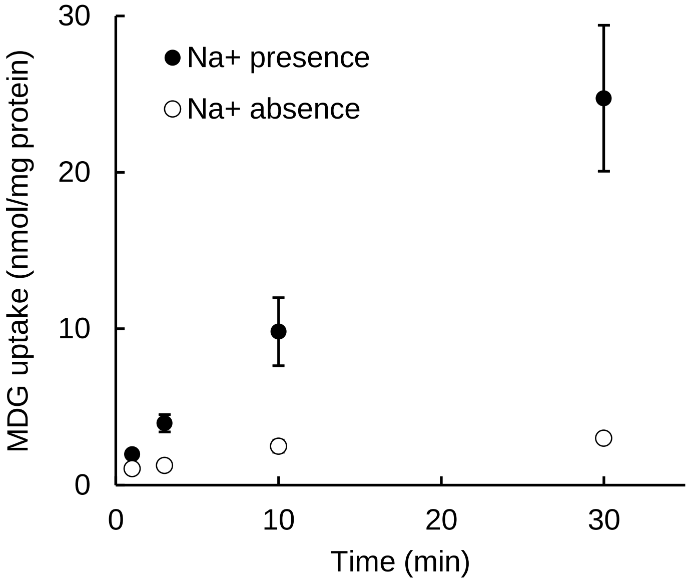

Supplementary Figure S1 Time-course of MDG uptake experiment

Uptake of MDG was increased in a time-dependent manner. The difference between them in MDG uptake between the presence of sodium ion and absence was as SGLT1 uptake.

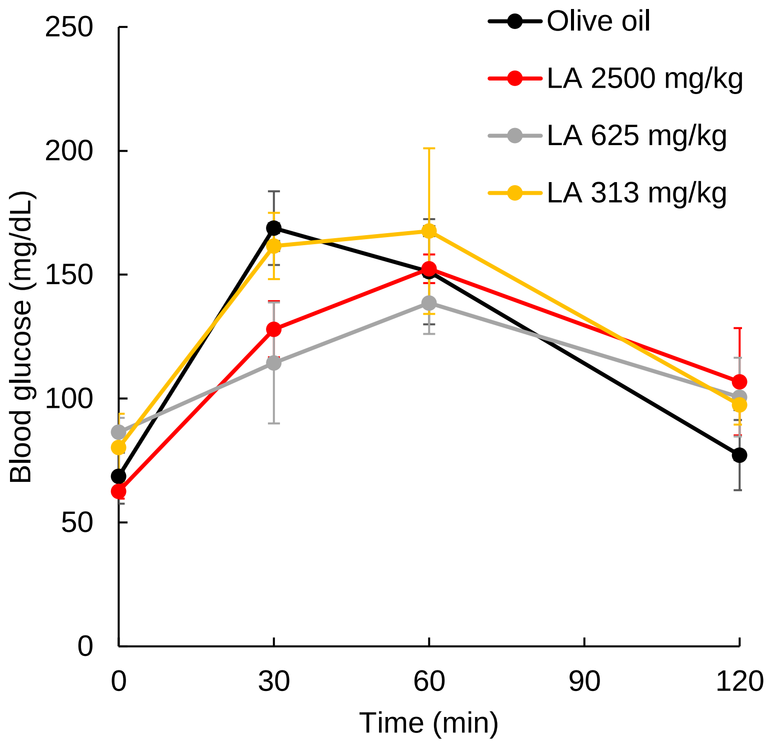

Supplementary Figure S2 Dose-dependent experiment in oral administration of LA immediately before glucose load

The slowing of elevation of postprandial blood glucose levels was induced by the oral administration of LA immediately before glucose load in dose-dependent manner.

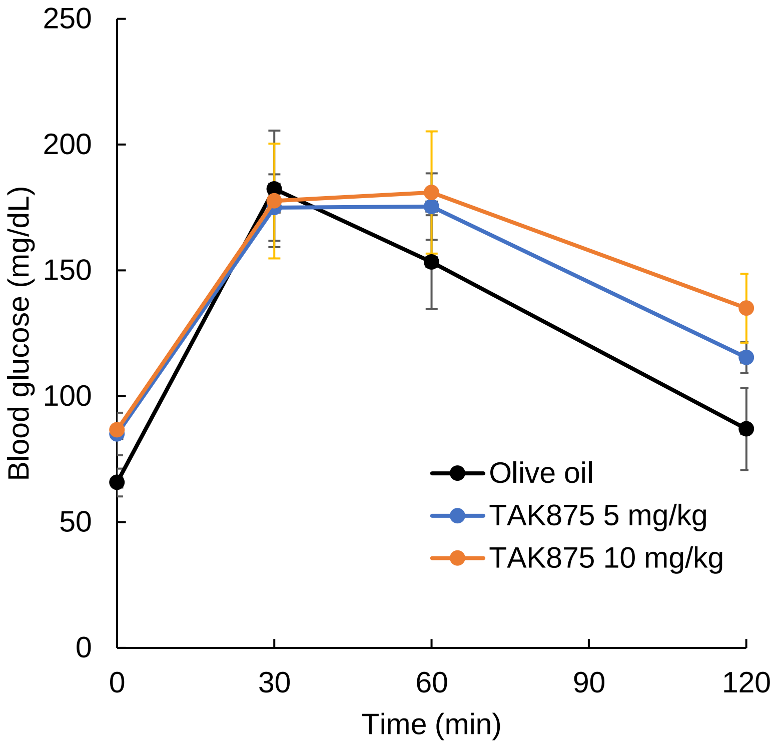

Supplementary Figure S3 Oral administration of TAK-875 immediately before glucose load did not slow the elevation of postprandial blood glucose levels.

Maintenance of postprandial blood glucose levels at high level was induced by the oral administration of TAK-875 immediately before glucose load in dose-dependent manner.
